# Comparative ethnobotany of the Wakhi agropastoralist and the Kyrgyz nomads of Afghanistan

**DOI:** 10.1186/s13002-015-0063-x

**Published:** 2016-01-06

**Authors:** Jens Soelberg, Anna K. Jäger

**Affiliations:** Museum of Natural Medicine, Faculty of Health and Medical Sciences, University of Copenhagen, Universitetsparken 2, 2100 Copenhagen, Denmark

**Keywords:** Afghanistan, Agropastoralists, Comparative ethnobotany, Kyrgyz, Medicinal plants, Nomads, Pamir, Traditional knowledge, Wakhan, Wakhi

## Abstract

**Background:**

The mountainous Wakhan and Pamir in northeastern Afghanistan is one of the most isolated yet inhabited places in Asia. It is home to the agropastoralist Wakhi and the last Afghan semi-nomadic Kyrgyz. We present a study of plant names and uses, along with comparisons of plant name etymology, origins of plant resources, intra- and intercultural exchanges and relations, and the relative availability of the known and used plants.

**Methods:**

The fieldwork was conducted as an expedition in the summer of 2010, and visited settlements and pastures in Upper Wakhan and Big and Little Pamir. Semi-structured group interviews, talks and observations gave initial data on names, uses and the relative availability of used plants, and provided foundation for individual interviews using an interview-herbarium containing vouchers of the 72 most frequently used plants or plant groups.

**Results:**

Wakhi and Kyrgyz plant names are recorded in western transcription, the new Wakhi alphabet, phonetically and in Cyrillic. The present study documents a large body of endemic, indigenous plant knowledge; on crops, fuel, fodder, cosmetics, dyes, vegetables, veterinary medicine, traditional medicines and other plant uses which sustain life in Wakhan and Pamir. Overall, the Wakhi use considerably more plants than the Kyrgyz, and their *materia medica* and use thereof is more complex. Although the Wakhi and Kyrgyz are close neighbours, there are few indications of direct knowledge transfer between them. Most shared plant uses are strictly necessary for survival in the mountains. While there are few differences between genders and cultural subgroups within the two cultures, the Wakhi and Kyrgyz exhibit great differences both in their total number of use-plants and the distance from which they obtain them. The agropastoralist Wakhi appear to have their basic needs for wild natural resources covered within half a days travel, while the relatively plant-derived environment of the high Pamir appears to have necessitated the nomadic Kyrgyz to adapt by developing uses and obtaining plants that are comparatively remote.

**Conclusion:**

The comparative differences in plant uses between the agropastoralist Wakhi and nomadic Kyrgyz appear to be accentuated by an environment at the extreme of what is humanly possible.

## Background

### Introduction

**Fig. 1:**
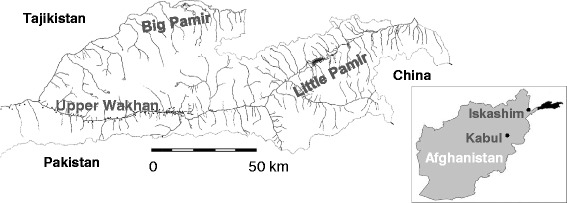
Map of the upper Wakhan valley and the Afghan parts of Little and Big Pamir

In the extreme northeast of Afghanistan lies the Wakhan District, which includes the Wakhan valley and the Big and Little Pamir (Figure 1). The Wakhan Corridor, as it is also known, is home to the entire population of two Afghan minorities: the agropastoralist Wakhi and the semi-nomadic Kyrgyz. The Wakhan Corridor is one the most isolated areas on the Asian continent, barred on three sides by closed international borders to Tajikistan, China and Pakistan. Being 2700 meters above sea level at its lowest altitude, it is an altogether mountainous area, and the meeting point of the Pamir Mountains, the Hindu Kush Range and other Himalayan ranges. The entrance to Wakhan and Pamir is through Iskashim in Badakshan, and the beginning of the Wakhan valley. Here live Wakhi farmers in permanent villages surrounded by irrigated fields, who, like their nomadic Kyrgyz neighbours, graze their livestock high in the Pamir pastures. The two Afghan Pamirs, Big and Little Pamir, are at their lowest altitude 4000 m.a.s.l. and is home to the Afghan Kyrgyz nomads. At the far east of the Wakhan Corridor are mountain passes leading to China, a part of the ancient Silk Road.

The first western account of the Wakhan Corridor was that of Marco Polo in 1273 [1]. In a scientific context, the first detailed ethnographic descriptions of the Wakhi and Kyrgyz peoples was those of Ole Olufsen, who lead the 1^st^ and 2^nd^ Danish Pamir Expeditions in 1896–97 and 1898–99 [2]. After Olufsen, there was limited research activity in Wakhan and the Afghan Pamir until the 1960’s and 70’s, which saw a small number of scientific and mountaineering expeditions. The most comprehensive work on Wakhi and Kyrgyz culture and their adaptions to closed frontiers, stems from this period [3].

The political borders of Wakhan and Pamir, drawn in 1895, initially meant little to the Wakhi and Kyrgyz, who moved freely across them for grazing and trade. However, as groups of Kyrgyz nomadic tribes in Russian-controlled territory witnessed the forced settling of their kin in present-day Tajikistan, they decided to stay permanently in their summer pastures in the Afghan Pamirs. Later, in 1949, the Communist revolution closed the border to China. Finally, movement over the border to Pakistan, which was previously permeable to trade and migrant work, has been greatly restricted in the aftermath of 9/11-2001. In a process far beyond the control of its inhabitants, the Wakhan Corridor has gone from being a highway of the Silk Road to a remote and confined cul-de-sac.

### The flora

Both Wakhan and the Afghan Pamirs present great contrasts in respect to vegetation; from vast stretches of cold desert to lush alpine meadows. Permanent snow is found everywhere above 5200 m.a.s.l., but the valleys receive less than 100 mm precipitation per year on average, and very little of it fall in the summer [2, 4]. Most running water in the valleys derives from melting snow and glaciers. The flora of the Wakhan Corridor has greatest relation to Central Asia and Tibet (compared to Persia, the Arctic and Kashmir), and some 20 % of the species are endemic to the area [5]. There are two broad ecological zones: alpine steppe and a temperate steppe environment. Within these zones are vegetation-types such as the sparse nival vegetation at the snow line, the alpine steppe with many aromatic perennials, alpine meadows with many showy herbs, tugai-vegetation in river plains, riverine forest along fast-rushing streams, temperate steppe and desert, patches of open woodland and shrub-land. Finally, the man-made irrigation systems create environments that allow for a relative profusion of herbs that are rarely or never seen in the rest of the Wakhan and Pamir.

### The Wakhi

The Wakhi people number around 50.000 individuals [6], an estimated 17.000 of which live in Afghan Wakhan. The Wakhi adhere to Ismailism within Shia Islam. They are likely descendants of Iranian tribes that arrived before AD, and like other Indo-Iranian mountain people of Afghanistan, some Wakhi have features such as blue eyes and blond hair. In Afghanistan, the Wakhi inhabit the entire Wakhan valley from its beginning outside of the town Iskashim to the easternmost village Sarhad. They speak *Xikwor* (generally referred to as Wakhi), a language belonging to the Indo-Iranian group, but all adults are bilingual, being also fluent in Dari. This bilingualism was already observed by a linguist in 1899 [7], who also stated that the Wakhi language was ‘evidently at the point of dying out’. Fortunately, this prediction was erroneous. Until recently, the Wakhi language was in Afghanistan purely oral, but sometimes written phonetically in Persian script [6]. In recent years a modified Persian alphabet is being employed in the many new schools in Afghan Wakhan.

The Wakhi are agropastoralist, living in villages and growing crops, as well as using high-altitude pastures for grazing livestock. They keep yak, cow, horse, donkey, goat and fat-tailed sheep. The upper altitude limits for wheat and barley are observed in the fields of upper Wakhan [6]. Wakhi agriculture is dependent on irrigation from melt water led through an often intricate network of small canals. Wakhi houses are built from timber, stones and mud-bricks, in a traditional style with a central living-room, an open fire-place, a smoke-hole, 5 pillars supporting the roof and raised platforms with rugs. The entire extended family lives in the central room, frequently surrounded by stables. In summers, the majority of Wakhi move to the pastures, either above the Wakhan valley on the Hindukush mountain slopes, or in Big and Little Pamir [3]. In the pastures the Wakhi live in simple stone-huts or yurts. In recent years, some Wakhi have changed their pastoralist system by keeping the animals on the pastures throughout the winter, with a small band of herders, only bringing down animals needed for consumption or sale.

A survey in 25 Wakhi communities in 2002 found that the most common causes of death reported were respiratory illness, gastrointestinal disease, measles, ‘other diseases’, bloody diarrhoea, chest pain, throat infection and diarrhoea. The under 5-year-old mortality rate was 314 in 1000 live births [8].

### The Kyrgyz

The number of Afghan Kyrgyz nomads was in 2010 estimated to be a total of 1253 individuals, 624 in Little Pamir and 639 in Big Pamir [9]. They are of paleo-Siberian origin and speak a dialect of Kyrgyz belonging to the Turkic language group. The Afghan Kyrgyz are Sunni Muslims. Most of the men are bilingual in Dari in the Big Pamir and Wakhi in the Little Pamir. They live in yurts, although some may have huts at their winter settlement. They move to and from fixed locations 3–4 times in a year, and are as such defined as semi-nomadic. They keep great flocks of fat-tailed sheep, as well as camel, yak, horse, donkey and goat. The Kyrgyz have no crops, and all essential food stock other than meat and dairy products are bought or bartered. Usually men will do one trip to Wakhan (and beyond) every fall, to trade and some years also to receive food aid. Most women, and those who do not travel due to trade or sickness, live continuously between 4–4.500 m.a.s.l. The seasonal movements between spring camps (*baaluu*), summer camps (*jailoo*), fall camps (*küzdöö*), and winter camps (*kyshtoo*) and pasture use, are governed by the concepts of *teskei (*the south-facing, sunny side of a valley) and *küngöi* (the north-facing, shady side of a valley) as part of a professed optimal grazing strategy [3, 9].

There is no formal schooling in Afghan Pamir. In a survey, which allegedly suffered from a relatively small sample size, 9 % of men and 6 % of women were literate in Big Pamir. The same survey found it difficult to determine the greatest health problems as the Kyrgyz responds often were vague. ‘Pain’ was most frequently cited, but also tuberculosis, measles, pneumonia, diarrhoea, high blood pressure and ‘fear’ was reported. The ‘under 5 mortality rate’ was estimated to be a dreadful 520 per 1000 live births [10].

### Prior ethnobotanical research

There have been no dedicated ethnobotanical studies done among the Wakhi and Kyrgyz. Prior published documentations of plant use in the Wakhan Corridor can be summed up in 3 points:

1) Wakhi agriculture: A description was given first by Olufsen [2] and Paulsen [11] and varies very little from later ones [3, 6]. 2) Wakhi uses of wild plants: Little has previously been reported besides the collection of an un-identified plant called *lakh* for soup in times of food-shortage [6]. Kassam [12] also reported various plants being collected in famine periods. Duncan and Duncan [8] reported that 70 % of the Wakhi use local herbs as medicinal remedies, and that those were “*jumbelak* for fever, *saosangul* for fever, *qaragat* and *puhdinna* (mint)”. 3) Kyrgyz uses of wild plants: Shahrani [3] noted collection of wood in lower Pamir for constructions of yurts, and gathering of a wild onion on high mountain passes as seasoning for meat. Duncan & Duncan [10] reported some use of herbs used for illnesses but were not able to obtain their names.

### Aims

The present study was conducted as a dedicated ethnobotanical survey with the aim to document all names and details of useful plants among the Wakhi and Kyrgyz nomads of Afghanistan, and to make this information available to the communities in written form. The study was designed to allow a comparative analysis of plant use and plant knowledge between groups defined by ethnicity, gender and geography, and relate these to the livelihood practises of agropastoralism and nomadism.

## Methods

### Field site

The fieldwork took place in June, July and August 2010, beginning in the Upper Wakhan valley (Wakhi villages), and proceeding to Big Pamir (Wakhi pastures/Kyrgyz settlements), over the mountain passes to Small Pamir (Wakhi pastures/Kyrgyz settlements) and returning to Upper Wakhan via Big Pamir. The last interview and plant collection took place on the 23^rd^ August at the arrival of the first snow. The expedition members were researcher Jens Soelberg, Wakhi interpreter Gorg Ali Khairkak and two Wakhi helpers; Jabor of Khandud and Jiama Göl of Baba Tengi.

### Plant collection

Plants were collected in triplets and deposited in the herbaria of University of Copenhagen, Botanical Museum (C) and Kabul University Faculty of Science (KUFS). For the 68 most widely used plants or plant groups the third specimen was inserted in an ‘interview-herbarium’ for use in semi-structured interviews. This herbarium/notebook is deposited at Museum of Natural Medicine, Copenhagen. Specimens of fungi and lichens, as well as samples of wood for documentation and anatomical determination of building materials, were also collected. Medicinal plant material and samples were either collected in the wild, bought or donated by respondents, and later deposited in the Museum of Natural Medicine, Copenhagen. Plant identification was based on Flora Iranica [13] and taxonomy updated according to www.theplantlist.org.

### Interviews

All interviews were assisted by the Wakhi interpreter Gorg Ali Khairkhak, who is tri-lingual in Wakhi, Dari and English. Interviews with Kyrgyz were conducted in their second language; Wakhi in Little Pamir, Dari in Big Pamir. Informed consent was obtained from individuals or community leaders orally while explaining the scope of the project prior to individual or groups interviews. Open-ended interviews, informal talks and plant-walks with both Wakhi and Kyrgyz supplemented the formal interviews.

#### Semi-structured group interviews

Semi-structured group interviews formed the first part of the data acquisition. At arrival in villages, after observing courtesies and practicalities, the present head-of-settlement was requested to gather as many men and women who could spare time. First, the assembled group was asked to name as many medicinal plants they could think of. When no more medicinal plants could be named, the group was asked to mention plants for treatment of sick animals, food, construction, fuel and other purposes, in that order.

To aid in assessing plant availability, a provisional ‘use-plant map’ was constructed by a show of hands of women and men in alternating sequence addressing the 3 questions: “*how many have been to X place* “, “*Did you bring plants from there*?”’ and “*If, which?”*. The map was plotted from near to far, beginning with the immediate surroundings of the village/settlement moving on to seasonal settlement, pastures, neighbouring settlements, neighbouring Wakhi or Kyrgyz territory and the bazaars of Khandud and Iskashim.

#### Semistructured individual interviews

Individual interviews was conducted using an ‘interview-herbarium’ and formed the second part of the data acquisition. From the group interviews, it was estimated that 72 plants or plant groups could be considered the most widely used. Vouchers of these were collected and mounted in random order in an A4-sized, hardcover notebook, and arranged ‘upside-down’ on the right page, allowing the interviewer to write information on the left page while the informant, sitting opposite or on the right-hand side, observed the voucher specimen. The respondents were asked to identify each plant in turn by the questions ‘*Do you know this plant?*’ or ‘*Does this plant has a name…?*’ Both positive and negative responses were noted. This was followed by questions detailing the use. Responses were noted or added to similar responses. For each respondent ethnicity (Wakhi/Kyrgyz), gender, age, settlement location and location of pasture(s) was noted. It was sought to get an even representation of four subcultural groups: Wakhi with pasture in Big Pamir, Wakhi with pasture in Hindukush and the two Kyrgyz groups of Little and Big Pamir. Each use of a plant or plant group presented in this paper was confirmed by at least three individuals, and each name confirmed by at least 10 individuals.

### Assessment of plant availability to sub-groups

Assessments of the relative availability of each of the 72 selected use-plants were made for the Wakhi and Kyrgyz geographical sub-groups. For each plant was assigned a value of 1 to 6 according to their abundance and geographical distance, measured in travel time by foot, in relation to the village or settlement. The assigned values were allocated by the researcher, based on the ‘use-plant maps’, field observations and interviews. The value “1” was assigned to plants being abundant and/or less than 1 h away from the group’s habitations. The values “2, 3 and 4” was assigned to plants occurring within a half-day’s travel from the group’s habitations, either common, less common or uncommon, respectively. Value “5” was assigned to plants only found more than one day away from group’s habitations; and “6” for plants only found more than several days away from the group’s habitations.

## Results and discussion

### Plant-name etymology

A small vocabulary of plant terms was assembled, a part of which is presented in Table 1. Wakhi, with linguistic origins in Persian, have plant-terms that come directly from Dari, such as ‘*daracht*’ (tree) or ‘*mihwah*’ (fruit), while others appear distinctly Wakhi such as ‘*sprech*’ (flower) or ‘*wus*’ (herb). The Kyrgyz of the Afghan Pamirs, who speak a language with Turkic roots, appear to have adopted some Persian terms such as ‘*mihwah*’ for fruit and ‘*bark*’ for leaf.

**Table 1:** Wakhi and Kyrgyz plant-terminology (D = Dari, W = Wakhi)

Term	Wakhi	Kyrgyz
Plant/herb	Wus	Ort
Tree	Daracht	Darak, Chöb
Leaf	Paltsh	Bark
Root	Berch	Tamyr
Stem/trunk	Destah	Djiratch
Branch/shoot	Zolgh	Shag
Flower	Sprech	Gül
Grass	Siwza	Kök
Fruit	Mihwah	Mihwah (D)
Seed	Tokhun	Ürön
Vegetable	Sawzi /Rasch	Sabzi (D/W)
Poison	Sar	Uuluu
Medicine	Dohrew	Dawa(D)/ Dary (W)

Both in Kyrgyz and Wakhi, as in English, the majority of plant names are proper nouns denoting a certain species or group of plants. Examples are the birch-tree (*Betula sp.*), which in Wakhi is ‘*förs*’ and in Kyrgyz ‘*kaii’in*’, or dandelion (*Taraxacum*), which in Wakhi is ‘*paps*’, or *Dracocephalum paulsenii,* which in Kyrgyz is ‘*marmuruu*’.

Wakhi have many compound plant names, often adding ‘-wus’ to denote the plant as a herb, as in ‘*gudunch-wus’* (*Elsholtzia densa*)*.* Also ‘*-dop’*, ‘*-pop*’ and ‘*-loy*’ may be added, but their meanings are unclear. ‘*Sprech*’ (‘flower’) is also commonly integrated into plant-names. One example of a morphological or taxonomical distinction integrated into a plant name is the adding of ‘*-shack*’ meaning ‘pea’ thus denoting the plant as pod-bearing legume, as in the wild ‘*liuw-shack*’ (‘crazy-pea’). Other examples of morphological plant-names are ‘*pai-sprech*’ (‘yogurt-flower’), which may encompass any small plant with a milky-white flower such as *Parnassia sp.* and *Cerastrium sp.,* or the common ‘*sart-sprech*’, meaning ‘yellow-flower’ is equivalent to the English vernacular ‘butter-cup’ in denoting any small, yellow, radial symmetrical flowered plant as those of *Potentilla* and *Ranunculus*. Compound plant names may be based on morphological features (as in ‘*sjars-wus*’, meaning ‘milk-herb’, which has a milky latex), their habitat (as in ‘*botch goez*’, ‘*goez*’ meaning meadow), their use (as in ‘*choi-wus*’, meaning’tea-herb’), resemblances to other plants (as in ‘*tjuan-wus*’, meaning ‘apricot-herb’) or animals (as in ‘*yuks-shack*’, ‘ibex-pea’, or ‘*chekör-dop*’, ‘partridge-weed’) or even combinations as in ‘*veengas goez*’, *Polygonum viviparum*, which translates into ‘meadow-sparrow’.

The Kyrgyz are equally sophisticated in their construction of plant-names. The addition ‘*oto*’ is the most common addition in denoting the plant as a herb, as in ‘*kii*’*ik oto*’, *Ziziphora clinopoides*. Morphological plant names occur as in ‘*tertri basch*’ (‘crooked head’) for a *Carex* with inclined flower heads. The name ‘*ular oto*’ is equivalent to the Wakhi plant name ‘*sart-sprech*’ and denotes plants with yellow, symmetrical flowers as in *Potentilla*. Another example of morphological plant names are those incorporating the word ‘*burma*’ meaning *‘drill*’. It is used in referring to plants with compound leaves and thus resembling a drill, as in ‘*kyzyl burma*’, meaning ‘red drill’ or ‘*burma qara*’ (*Zygophyllum obliquum*) meaning ‘black drill’. There are many examples of plants named in resemblance to animal parts, as ‘*tör kuirik*’, meaning ‘camel-tail’, a legume, or ‘*at tepik*’, meaning ‘horse-penis’, referring to mushrooms. ‘*Tiken*’ or ‘*sadé*’ might be added to a plant name to denote its growth in either groups or singly, or possibly the growth form, having one or many stems. Some plants are named after people and/or their use as in ‘*Kolikae-chai*’, *Potentilla bifurca*, ‘*Kolikae*’ apparently being a man’s name and ‘*chai*’ meaning ‘tea’. The aromatic herb *Dracocephalum paulsenii* is called ‘*marmuruu*’ or ‘*erkik marmuruu*’, ‘*erkik*’ meaning ‘female’. A ‘male’ type*, ‘urganchy marmuruu*’, also exists, and may refer to the relatively rare, white-flowered variety of *D. paulsenii*.

### Completeness of study

In total 140 adult Wakhi men and 80 women were interviewed, corresponding to 1.3 % of the entire population of approx. 17.000 individuals. Of Kyrgyz, 61 men and 9 women were interviewed corresponding to 5.6 % of the entire population of 1253 [9]. In Wakhi group interviews the average number of participants were 10.5, and the men to women ratio was near 2:1. The gender ratio was nearly the same in both villages and pasture settlements. Among the Kyrgyz the average number of participants were 4.5, and men to women ratio was 6.6:1.

**Fig. 2:**
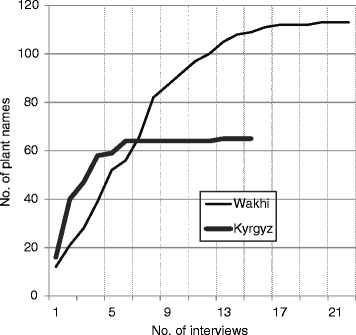
Completeness of study

Between the interviewed Wakhi communities some 113 names of useful plants or plant-groups were noted. By the 22^nd^ interview the list of useful plants was considered to be complete, as the later interviews did not add further plant names (Fig. 2). Between the Kyrgyz communities interviewed, 65 names of useful plants were noted and the list was considered complete by the 15^th^ interview (Fig. 2).

The list of wild useful plants was reduced from a total of some 150 plants or plant-group names to 72 representative use-plant species or species complexes. These reductions were mainly done by grouping plants (such as several species of *Salix, Acantholimon* and *Artemisia*).

### Uses of plants

The uses of wild plants by Wakhi and Kyrgyz was divided into 10 groups: fuel, fodder, construction, cosmetics, dye, tea-substitutes, vegetables, incense, veterinary medicine and medicine. The number of plants falling into each of these use-categories is summed up in Table 2. For the full list of wild plant names and uses documented among the Wakhi and Kyrgyz, see Table 3.

**Table 2:** Number of plant species or groups within use-groups

Usegroups	Number of plants used by Wakhi	Number of plants used by Kyrgyz	Total
Fuel	11	7	12
Fodder	6	6	8
Construction	14	9	13
Cosmetics	10	1	10
Dye	3	1	3
Vegetables	16	7	16
Tea-subst.	7	4	7
Incense	4	2	4
Vet. med.	11	5	15
Medicine	32	15	36

**Table 3:** Plants and plant groups used by the Wakhi and Kyrgyz

Names	JS#	Wakhi use	Kyrgyz use	Notes
Fungi **Basidiomycetae** Voucher: ***Agaricus sp.*** W: Lehwund/ le'vund / لیقوند K: At Tepik/ ат тепик	NMM		**Veterinary:** Spores applied to infected animal wounds 'to dry them out'.	Not eaten anywhere in Wakhan. By the Kyrgyz considered 'unclean'. Large fruiting bodies are not common in the dry climate of Wakhan. Etymology: *At Tepik* (K) means 'horse-penis'.
Lichen **Teloschistaceae** Voucher: ***Xanthoria elegans*** (Link) Th.Fr. W: Rabooch/ɾa’bɔtʃ / رباچ K: Engelchek/ энгелчек	NMM	**Veterinary:** Ground and applied to infected wound of both human and animal.	**Veterinary:** Ground and applied to animal wounds. **Veterinary:** Ground, mixed in butter, fed to animals with diarrhoea, esp. yak-calves.	Lichens are common everywhere in Wakhan. No species or colour is preferred in use. Etymology: Ra-booch (W) means 'stone-dirt'.
Moss **Bryaceae.** Voucher: ***Bryum sp***. W: Khor-döb/ rrəɾ’ðʊp/ خردپ K: Balohr/ балэр	285	**Veterinary:** Inner bandage on animal wounds **Medicine:** Eye-bandage.	**Veterinary:** Inner bandage on animal wounds.	
***Allium spp.*** **Amaryllidaceae** W1: Lönntörk/ لنترک W2: Shög-shög/ شږشږ K1: (Sasyk) Dana/(сасык) дана K2: Köbürgön/ көбүргүн	28; 101; 224	**Vegetable:** Vegetable and flavour, often dried for later use. **Medicine:** Used in food for feverish patients. Generally eating *Allium* is considered good for health. **Medicine:** *Lönntörk* (W) is boiled, water discarded, and the remaining plant parts mixed with flour and eaten for diarrhoea.	**Vegetable:** Vegetable and flavour, often dried for later use. **Medicine:** Used in food for feverish patients. Generally eating Allium is considered good for health. **Medicine:**Dana and Sasyk-Dana is boiled and the decoction applied to itching rashes.	There is quite a diversity of onions and their uses. There is a tendency for the finer, drooping ones to be known as *Shög-shög* (W)/*Köbürgön*(K), and the sturdier, upright ones as *Lönntörk* (W)/*Dana*(K). Most medicinal uses were associated with the sturdier ones. Etymology: *Sasyk* (K) means 'bitter'.
***Carum carvi*** L. **Apiaceae** W: Nurtök/ nur’tʊk/ نرتک	17	**Vegetable:** Seeds as spice. **Vegetable:** Herb eaten fresh when young. **Vegetable**: Seeds eaten fresh and dried. **Medicine**: In food for feverish patients. **Medicine:** Decoction of fresh or dry seeds for throat-pain.		*C. carvi* is naturalised around Wakhi irrigation-canals and not used by Kyrgyz. It is likely that the appearance of *C. carvi* is similar to the plant known to Kyrgyz as *tiken kai'sar*.
***Anaphalis virgata*** Thoms. **Asteraceae** W: Yipén /’jipən/ هیپن K: Aq Rasul/ ак расул	135	**Medicine:** Decoction drunk for fever and breathing trouble (will cause sweating). **Miscellaneous:** Put with wrapped bread for the bread to keep fresh **Incense:** Incense. **Fuel.**	**Medicine:** A 'double decoction' of the herb is drunk for high blood pressure and heart-pains.	This is one of the few medicinal plants the Kyrgyz admitted to bring from Wakhi territory. Etymology: *aq rasul* (K) means 'white prophet' or 'white messenger'.
***Artemisia spp.*** **Asteraceae** W: Tebesk/ tə’bəsk/ تبسک K: Suak/ суак	n/a	**Fuel:** Firewood and kindling. Wakhi distinguish *mai*-(sheep), *seuv*-(black), *rochun*-(white) and *trök-tebesk*. **Construction:** Used as insulating and/or ventilating layer in roof-construction.	**Fuel:** Firewood and kindling. Kyrgyz distinguish *koi*-(sheep), *kara*-(black), *sary*-(yellow, *aq*-(white) and *kok*-(wool)-*suack*.	*Artemisia*-shrubs are only collected for fuel, and not fodder. However, few herders told about their animals' preferences in fresh *Artemisia-shrubs*.
***Artemisia dracunculus*** L. **Asteraceae** W: Sars/ saɾs/ شرث	146	**Medicine:** Leaves, flowers or flower buds toasted lightly and applied to swollen body parts. **Fodder.**		
***Artemisia persica*** Boiss. **Asteraceae** W: Rahweet/ ɾa’vet/ رفید K: Ermin/ эрмин	154	**Medicine:** Herb boiled, mixed with butter and applied to chest for common cold. **Medicine:** Leaves and flowers toasted, applied to swollen body parts. **Medicine:** Decoction drunk for stomach trouble and/or headache. **Medicine:** Juice from pressed herb applied to face to shun coldness. **Dye:** Clothes or wool boiled with herb for yellow dye. **Tea-substitute.**	**Medicine:** Rashes washed in decoction from herb. **Medicine:** Decoction drunk with milk for headache.	The fresh herb has a camphoric smell.
***Artemisia sieversiana*** Willd. **Asteraceae** W: Setwörk/sət’wəɾk/ ستوورگ	152	**Medicine:** Dry herb crushed, applied to burns. **Miscellaneous:** Fresh herb placed with stored rugs, wool or yarn to keep off moths. **Veterinary:** Dry herb applied to maggot-infested animal wounds.		
***Berberis heterobotrys*** Wolf. **Berberidaceae** W1: Zolg/ zɔlg/ زلگ W2: Karakot/ kara’qɔt/ قره قات (the fruit). W3:Zar-ruhl/ zər’rrul/ زرغول (the root)	172	**Medicine:** For heart problems and high blood pressure. Dried fruits placed in water for few minutes, then squeezed by hand. The cold-extract is filtered, mixed with sugar and bottled for regular use. **Medicine:** Decoction drunk, or dry pulverized root eaten, for parasites and stomach problems. **Veterinary:** Decoction of the yellow root given to weak oxen and horses in spring.	**Medicine:** Dried fruits decocted, drunk for fever (rare use).	Etymology: z*ar-ruhl* is taken from Dari (Persian) and may be translated 'bitter-fool'.
***Betula chitralica*** Browich **Betulaceae** W: Förs / fərs/ فرز K: Kaii'ing/ каийиң	36	**Medicine:** Lightly toasted bark as wrapping for broken finger, toes, etc. **Medicine:** Bark crushed on burns. **Medicine:** Prayers written on bark by a Mullah, subsequently a decoction is made and drunk for various diseases. **Construction:** General construction. **Construction:** Branches used as brooms. **Miscellaneous:** Bark traditionally used like paper.	**Construction:** Construction of the *tündök*, the crown piece of the yurt.	
***Arnebia guttata*** Bge. **Borraginaceae** W: Pusch/ pʊʐk/ پشک K: Endik/ эндик	145	**Medicine:** Root chopped finely and fried in oil, strained and applied to children's ear ache, with or without visible inflammation. **Cosmetic:** Fried root-oil used for dry hair and dandruff. **Dye:** Intense red or purple.	**Dye:** Root collected for red dye. **Cosmetic:** Dye used in women's cosmetic.	
***Myosotis asiatica*** Schisck. & Serg **Borraginaceae** W1: Kohl-wus/ qəl’wʊɛ/ قال وښ. W2: Sawsan-sprech/ Saʊsan sprəʊɟ/ سوسن سپږ	118	**Cosmetic:** Flowers used fresh, cold or warmed, or dried and later soaked, as skin cream by mainly women.		Etymology: kohl (W) means cream. Note: sawsan-sprech also refers to *Primula pamirica*, and the uses are similar.
***Brassica napus*** L. **Brassicaceae** W: Cheror/ tʃə’rɔrr/ چراغ	14	**Vegetable:** Leaves collected for food throughout spring. **Cosmetic/Miscellaneous:** Seeds toasted and ground to a paste, a moisturizing and heat preserving skin cream used in cold weather. **Veterinary:** Seeds ground and fed to calves with constipation.		Traditionally, seeds were the sole source of oil for lighting.
***Descurainia sophia*** (L.) Webb & Berth **Brassicaceae** W: Kehskritsch/ kəɛ’kɾitʃ/ کشکریچ	151	**Medicine:** Seeds ground, mixed with water to paste, applied to blisters, rashes and swellings. Sometimes drunk additionally. **Medicine:** Seeds ground to powder, blown with pipe into throat of a person suffering from throat pain. **Medicine:** Decoction of seeds drunk with or without milk for constipation.		
***Lonicera asperilifolia*** (Decne) Hook, f & Thomas **Caprifoliaceae** W1: Chöpar-push/ tʃə’parpʊɛ/ چپر پوش W2: Speen/ ʃpin/ سپین K: Schilvi / шилви	138	**Construction:** Valued wood used for dowels, spindles, and other tools. **Cosmetic:** Fruit used fresh (or kept) as moisturising skin cream. **Fuel.**	**Construction:** Wood to construct wool-spindles and whips. **Construction:** Nose-wood for camels and yak. **Miscellaneous:** Bark used as sponge in cleaning.	*Chörpar-push* and *speen* (W) are intermittently used for this spiny *Lonicera.* The non-spiny *L. pamiric*a is only called speen.
***Lonicera pamirica*** Pojark. **Caprifoliaceae** Cfr. ***L. microphylla*** Willd. W: Speen/ ʃpin/ سپین	139	**Construction:** Valued wood used for dowels, spindles, and other tools. **Cosmetic:** Fruit used fresh (or kept) as moisturising skin cream. **Fuel.**	**Construction:** Valued wood used for dowels, spindles, and other tools. **Cosmetic:** Fruit used fresh (or kept) as moisturising skin cream. **Fuel.**	This is the over-all preferred skin moisturizer among the Wakhi. Everyone is familiar with it, but it is generally only used by women.
***Silene conoidea*** L. **Caryophyllaceae** W: Peetpetak/ pit pə’tak/ پیټ پی ټک	153	**Vegetable:** Seeds eaten enthusiastically by Wakhi children.		
***Vaccaria grandiflora*** (Fisch. ex DC) Jaub & Spach. **Caryophyllaceae** W: Serah/ ɛər’rra/ څیرغه	149	**Miscellaneous:** Soap, fresh, rubbed with clothes. **Miscellaneous:** Mixed in grain before milling to produce whiter flour.		
***Chenopodium album*** L. **Chenopodiaceae** W: Schlit/ ʃlit/ ثیلټ K: Shakar/ шакар	15	**Vegetable:** Collected as vegetable throughout spring and early summer.	**Fodder:** Collected for fodder.	The same use apply to *C. glaucum* and *C. pamiricum*.
***Chenopodium botrys*** L. **Chenopodiaceae** W: Ziuck/ si’ʊk/ سیوگ (K: Surak/ сурак)	150	**Vegetable:** Used as vegetable, but boiled first and water discarded, to prevent bitterness. **Tea:** Tea-substitute.		*Surak* (K) was only named few times by Kyrgyz; it might be a misidentification of this aromatic plant mainly found in Wakhi villages.
Unidentified **Chenopodiaceae** (W: Schlit/ ʃlit/ ثیلټ) K: Shakar/ шакар	211	(**Vegetable:** It is uncertain whether this plant is collected, but it resembles *schlit (Chenopodium album)* which is.	**Miscellaneous:** Ashes of herb ground finely, boiled with water and oil or fat to produce soap.	This is the only apparently anthropogenic plants in the high Pamirs. It is only found on heavily fertilized Kyrgyz winter camp grounds.
***Krascheninnikovia ceratoides*** (L.) Gueldenst. **Chenopodiaceae** Syn: ***Eurotia ceratoides*** (L.) C.A.Mey.W: Schitten/ ’ʃitən/ شیتن W2: Tishkan/ tis’kən/ تیشکن K: Tersken/ терскен	6	**Fuel:** Important firewood. **Miscellaneous:** Ashes used as an ingredient in snuff (*naswar*), but only when *E. intermedia* is not available.	**Fuel:** Important firewood. **Miscellaneous:** Ashes used as an ingredient in snuff (*naswai*).	*Schitten* and *tishkan* is the Wakhi name for the same plant in Wakhan or Pamir respectively. It is often less compact and less 'woolly' in Wakhan. The Kyrgyz call particularly dense-haired specimens *kok*(wool)*-tersken*.
***Rhodiola heterodonta*** (Hook. f. & Thoms.) A. Boriss. **Crassulaceae** W: Ströj-rölöy/ ستر یغلای	50	**Medicine:** Decoction of herb, fresh or dried, for stomach ache. **Medicine:** Herb in food against 'blood-accumulation'.		Etymology: *ströj* (W) means female or she.
***Juniperus excelsa*** M. Bieb. **Cupressaceae** W: Yarz/ jarz/ یرز K: Archa/ арча	133	**Medicine/Incense:** Soothing incense for the sick. **Medicine:** Decoction of leaves drunk against 'liver-worms'. **Miscellaneous:** Ashes of branches used as lice-disinfectant. **Veterinary:** Coal of wood ground and applied to animals' eyes when gone blind from eating *kumut* (W), *Tetrataenum olgae*. **Construction:** General construction. **Construction:** Construction of instruments. **Miscellaneous:** Branches carried when visiting neighbours and relatives during Nauroz (New Year).	**Incense:** Soothing incense for the sick. **Construction:** Construction of tools.	
***Carex sp.*** **Cyperaceae** W1: Shuber-wus/ ’ʃəbeɾwʊɛ/ شبر وش W2: Botch goez/ بج گاز K1: Tertri bash / тетри баш K2: Suerh/ шуубер	207	**Fodder:** Collected as winter-fodder for animals.	**Fodder:** Collected as winter-fodder for animals.	
***Hippophaë rhamnoides*** L. **Elaeganaceae** W1: Zach/ zaç/ زڅ W2: Khos-gök/çoɛ’gʊk/خوڅ گگ (the fruit) K: Checher Kana/ чечер кана	157	**Medicine:** Fruits decocted, filtered and bottled. Drunk in mornings for joint pains. **Medicine:** Decoction of fruits drunk for jaundice. **Cosmetic:** A sun-protection skin cream is made from berries. **Construction:** General construction. **Fuel:** Firewood.		*Khos-gök* (W) is the name of the orange, sour berries. Abundant in the Wakhan river valley.
***Ephedra intermedia*** Schrenk & Mey. **Ephedraceae** W: Ihmück/ ’imək/ ایمک K: Chekender/ чекендер K2: Chechender/ чечендер	163	**Miscellaneous:** Ashes of shrub mixed with tobacco to produce snuff (*naswar*). **Medicine:** Dislocated joints re-set while submerged in bath of decoction. **Medicine:** Steam-bath or footbath for aching feet/legs. Medicine: Mouth-wash for tooth-ache. **Miscellaneous:** Yogurt-starter.	**Miscellaneous:** Ashes of shrub mixed with tobacco to produce snuff (*naswai*). **Medicine:** Green parts ground and applied to swollen stomach or aching shoulder/back. **Medicine:** A paste made from green parts rubbed on chest of people with 'fear' (anxiety or other psychological disorder).	
***Ephedra regeliana*** Florin **Ephedraceae** W: Ihmon-ihmück/ i’mɔn ’imək/ ایمان ایمک K1: Chekender/ чекендер K2: Chechender/ чечендер	140	**Vegetable:** Berries eaten.	**Vegetable:** Berries eaten.	
***Cicer microphyllum*** Benth. **Fabaceae** W: Pisch-wus/ ’piʃwʊɛ/ پیش وش W2: Yuks-shack/ jʊkɛ-ɛaç/یوک شڅ. K: Tash Kurut/ таш курут	35	**Vegetable:** Flowers and seeds eaten. **Construction:** An alternative material in Wakhi layered roof building. **Miscellaneous:** Yogurt-starter.	**Vegetable:** Flowers and seeds eaten. **Medicine:** Flowers eaten for altitude sickness. **Fodder:** Collected for animals as fodder.	Etymology: *pisch*-wus and *yuks-shack* (W) is used intermittently. They mean 'cat-herb' and 'ibex-pea' respectively. The pod curls to resemble ibex-horns when dry.
***Glychyrrhiza uraliensis*** L. **Fabaceae** W: Matk/ matk/ متک K: Scherin-buya (Dari origin)	156	**Dye:** Boiled with yarn or wool to produce yellow colour. **Miscellaneous:** Root chewed for the pleasant taste. **Construction:** Component in Wakhi layered roof building. **Veterinary:** Decoction given to nursing cows to improve milk quality. **Fodder.**		Some Kyrgyz know this plant by its Dari (Persian) name. It does not grow in the high Pamir.
***Astragalus sp.*** **Fabaceae** W: Purk-zach/ pʊrk-zaç/ پرک زښ	32	**Miscellaneous:** Rinsed root is used for tooth-cleansing in the manner of a toothbrush.		Etymology: *purk-zach* either means 'mouse-pea' or 'mouse-thorn'. It has long, flexible thorns and is used by Wakhi to seal mouse-holes.
Unidentified **Fabaceae** K: Tör-kuiruk	200		**Construction:** Broom material. **Fuel:** Firewood.	Etymology. *tör-kuiruk* (K) means 'camel-tail', which the dug-up plant indeed resembles.
Unidentified **Fabaceae** W: Chekör-dop/ tɛəkər’ðɔp/ څکر قاپ K: Nokhetek/ нохетек	199	**Fodder.**	**Fodder.**	There is no consensus among Kyrgyz or Wakhi herders whether this plant is poisonous when wet, when dry, when the animals are weak in the spring, in too large quantities etc., but all agree that under wrong circumstances it may be poisonous.
***Ribes villosum*** WaU. **Grossulariaceae** W: Chöllasm/ tɛə’laʐəm/څلږم K: Qaragat/ карагат	3	**Vegetable:** Berries eaten. **Fuel:** Firewood.	**Vegetable:** Berries eaten.	Supposedly, another variety of *Ribes* with aromatic leaves also occurs.
***Dragocephalum paulsenii*** Briq. **Lamiaceae** W1: Choi-wus/ tʃɔi-wʊɛ/ چای وش W2: Wazir-choi K1: Marmuruu / мамуруу K2: Erkek Mamuruu/ эркек мамуруу	51	**Tea:** Tea-substitute.	**Medicine:** Decoction for fever. **Medicine:** Decoction for altitude-sickness. **Tea:** Tea-substitute.	Kyrgyz distinguish *erkek* (female) *mamuruu* and *urganchy* (male) marmuruu. The voucher had blue-violet flowers, but a white type was observed, however seldom, and might be the 'male'-*marmuruu*.
***Elsholtzia densa*** Benth **Lamiaceae** W: Gudunch-wus/ gʊ’dʊnʃ- wʊɛ/ گدوندچ وش	147	**Medicine:** Decoction drunk +/- milk for common cold, fever, headache, stomach problems, joint ache and high blood pressure. **Cosmetic:** Herb crushed and put in direct sun for few minutes, pressed for juice to make a skin and facial cream by women. **Tea-substitute:** Tea-substitute and flavour-adder to cold water.		
***Lagochilus cabulicus*** Rech, f & Edelb s.l. **Lamiaceae** W: Sjus-wus/ ɛʊɛ- wʊɛ سنوښ وش	141	**Medicine:** decoction given to animals with respiratory problems.		Etymology: *sjus* (W) means lung.
***Mentha longifolia*** (L.) Hudson **Lamiaceae** W: Wadén/ ’wadən/ ودن	175	**Medicine:** Fried and eaten for fever. **Medicine:** Decoction commonly drunk for variety of diseases and maladies. **Medicine:** Eaten dry with sugar for various diseases. **Veterinary:** Fed to calves and yak with skin problems, 'sun-burns'.		Note: Some Wakhi women believe use of this plant may cause infertility.
***Nepeta pamiriensis*** Franch. **Lamiaceae** W: Mai-mendritsch/ mai-mən’dritʃ / می مندریتچک K: Boznoch/ бозноч	125	**Medicine:** Decoction drunk +/- milk for fever, nausea and various unwellnesses. **Tea-substitute.**	**Medicine:** Decoction drunk +/- milk for fever, nausea and high blood pressure. **Medicine:** Used in food of feverish patients. **Tea:** Tea-substitute.	
***Ziziphora clinopoides*** Lam. **Lamiaceae** W: Jumbilack/ dʒʊmbi’lak/ جمبیلک K: Kii'ik Oto/ кийик ото	107	**Medicine:** Decoction +/- milk drunk for altitude-sickness, headache, respiratory problems, and heart trouble. **Tea:** Tea-substitute and flavour-adder to cold water.	**Medicine:** Decoction drunk +/- milk for heart trouble and high blood pressure. **Medicine:** Leaves eaten fresh for heart troubles. **Tea:** Tea-substitute.	The Kyrgyz of Big Pamir know and use this plant, but many Little Pamir Kyrgyz neither recognise or of know it, though equally common. One old Kyrgyz has observed wild animals self-medicate with it.
***Epilobium latifolium*** L. **Onagraceae** W: Sorch-sotsch/sʊrχ-sɔtʃ / سرخ ساچ	136	**Medicine:** Applied fresh, ground or as a paste-like boiled-down decoction to swollen legs and leg-pains. Decoction may be drunk additionally. **Medicine:** Herb ground to paste, fresh or dry with water added, applied to blisters and abscesses, believed to 'exude pus'.		
***Papaver involucratum*** Popov. **Papaveraceae** W: Göl-mervoi/ gʊlmər’wɔi/ گل مروای	288	**Miscellaneous:** All Wakhi light up in a smile by the mentioning of this plant and seem to enjoy both word and sight.		
***Plantago spp.*** **Plantaginaceae** Voucher: ***Plantago gentianoides subsp. griffithii*** (Dechne.) Reich. W: Setbilk/ ست بلیگ / sət’bilk	16	**Medicine:** Fresh leaves bruised, or leaves or seeds ground to paste, applied to blisters, abscesses and wounds. **Medicine:** Leaves in food or fried for constipation, joint pains and 'springtime respiratory problems'.		
***Acantholimon sp.*** **Plumbaginaceae** W: Altpök /alt’pək/ التپک K1: Kurtka/ куртка K2: Kyzyl Tiken/ кызыл тикен	191; 292	**Fuel:** High-altitude firewood.	**Fuel:** High-altitude firewood.	*Schöt-altpök* (W) is a smaller species, often found in sand, and less useful as fuel. *Schöt* (W) means sand.
***Calamagrostis pseudophragmites*** (Hall, f. ) Koeler **Poaceae** W: Chörr/ tʃərr/ چغ K1: Kyak/ кыак K2: Cher/ чер	21	**Construction:** Material for screens, baskets, brooms. **Construction:** Element in layered roof building. **Medicine;** Stem quickly passed through fire before rubbed on various skin problems. **Medicine:** Mid-part of stem of ungrazed plants decocted and drunk for jaundice, bloody vomit and bloody diarrhoea. **Fodder.**	**Construction:** Material for screens used for insulation and room separation in yurt. **Fodder.**	
**Poaceae** Voucher: Unidentified Poaceae W: Spöd/ ʃpɔd/ شپد K1: Gödö/ гөдө K2: Betege/ бетеге	202	**Fodder.**	**Fodder.**	A number of grasses are known by name, nutritional quality, etc. by Wakhi and Kyrgyz herders.
***Oxyria digyna*** (L.) Hill. **Polygonaceae** W: Thresp-pop/ trəɛp-pɔp/ ترښپاپ K: Kozu Kulak/ козу кулак	55	**Vegetable:** Leaves eaten fresh. A valued thirst-quencher on journeys and while herding. **Miscellaneous:** Yogurt-starter.	**Vegetable:** Leaves eaten fresh.	Etymology: *Thresp* (W) means 'sour'.
***Polygonum spp.*** **Polygonaceae** Voucher: P. viviparum L. W: Veengaz-goez/ wingas ’gɔz/ وینگس گاز K: Jörgömüsh/ жөргөмүш	25	**Vegetable:** Seeds eaten fresh. **Vegetable:** Root (Pud-goez/ pʊd gɔz/ پوټ گاز ) is eaten fresh.	**Vegetable:** Seeds eaten fresh. **Vegetable:** Root (Mandalak/ мандалак) is eaten fresh. **Vegetable:** Seeds mixed in cream for flavour and texture.	Etymology: *veengaz* (W) means 'sparrow', *goez* (W) refers to 'meadow'. There may be other species within the genus used similarly.
***Rheum spiciforme*** Royle **Polygonaceae** W: Spod/ ʃpɔd/شپاد K: Chükürü/ чүкөрү	57	**Vegetable:** Lower leaf stalk eaten fresh. **Miscellaneous:** Thirst-quencher on journeys.	**Vegetable:** Lower leaf stalk eaten fresh. Lower flower stalk (ushky/ ушку) are equally enjoyed.	At times collected and carried over distances for refreshment and thirst-quenching. Highly praised by all Wakhi. The Spodkis-valley is named after this rhubarb.
***Rumex spp***. **Polygonaceae** Voucher: ***R. patienta ssp. pamiricus*** Rech. W: Schellkha'/ ʃəl’χa/ شلخه	161	**Medicine:** Used in a kind of moxibustion, in which small scrapings of stem is placed on the skin at a place of joint, skeletal or muscular pain and lit a-fire. May leave small scars. **Medicine:** High-fever patients are wrapped in wet leaves, which may additionally be stuffed into pillow and quilt. **Medicine:** Leaves in food for fever patients. **Medicine:** Decoction of leaf or root for fever. **Medicine:** Root-decoction drunk for constipation. **Veterinary:** Root ground and applied to animal wounds.		
***Primula macrophylla*** Don. **Primulaceae** W: Benafsch/ bənafʃ/ بنفش K: Benavsh/ бенавш	114	**Medicine:** The white farinose powder on leaves and calyx, referred to as 'dust', *gart* (W), is applied directly on eyes suffering from pain and blurred vision due to years of exposure to smoke and strong sun-light. The 'dust' is beaten off and applied directly to the eyeball with a soft-stone stick, known as a *punsk* (W). Alternatively, the plants are steeped in water causing the 'dust' to surface. It is then skimmed off, boiled-down into a transportable lump, and applied to eyes by means of the *punsk*.		The use of *P. macrophylla* is known by all Wakhi, but mainly used by elderly. Interviews with Wakhies from communities in Pakistan and Tajikistan indicated the same or similar use there. Only few Kyrgyz know of the Wakhi use.
***Primula pamirica*** Fedor. **Primulaceae** W: Sawsan-sprech/ Saʊsan sprəʊɟ/ سوسن سپږ	38	**Cosmetic:** Fresh flowers ground by hand and applied as a skin cream on face and hands, mainly by girls and young women. **Medicine:** Decoction of herb drunk for heart and respiration troubles.		
***Delphinium spp.*** **Ranunculaceae** Voucher: ***D. brunonianum*** Royle W: Ambar/ am'bar/ عمبر K: Jal Byrak/ жал бырак	113	**Medicine:** Incense for the sick and to calm children. **Cosmetic:** Heat-extracted in oil or fresh as a paste against dandruff. **Veterinary:** Herb ground and applied on maggoty animal wounds.	**Veterinary:** Decoction of herb boiled-down to paste, as an ointment for cuts and wounds.	A few *Delphinium* species occur in Wakhan and Pamir. *Ambar* supposedly only refers to the aromatic one(s). A popular poem and song in Badakshan celebrates 'the *ambar* of Pamir'.
***Pulsatilla campanella*** Fisch **Ranunculus** K: Kundus-kayer/ кундус каер	246		**Veterinary:** Herb ground and applied to infected animal wounds.	
***Ranunculus*** spp. **Ranunculus;** ***Potentilla spp.*** **Rosaceae** Voucher: ***Potentilla pamiroalaica*** Juz. W: Sart-sprech/ zart-sprəʊɟ/ ارت سپرژ K: Ular Oto/ улар ото	127	**Cosmetic:** Fresh flowers pressed for juice which is applied to cracks and crevices in dry skin. This is one of the few 'cosmetic' plant uses men will admit to use. One man noted that only the smaller, delicate herbs (likely *Ranunculus sp.*) were the correct to use.		Both Wakhi and Kyrgyz group yellow-flowered herbs into *sart-sprech* (W) and *ular oto* (K), for which the English equivalent would be 'buttercups'.
***Potentilla bifurca*** L. **Rosaceae** W1: Morsjönd W2: Choi-wus/ tʃɔi-wʊɛ/چای وش K1: Kyzyl-chai/ кызыл чай K2: Kolikae-chai/ кoликай чай K3: Tannu-chai/ танну чай	176	**Tea:** Tea-substitute.	**Tea:** Tea-substitute.	Kyrgyz key informants informed that all three names can be used in parallel. *Kyzyl* (K) means 'red'. *Morsjönd* (W) is prevalent in upper Wakhan, whereas *choi-wus* (W) is more common in lower and middle Wakhan.
***Rosa webbiana*** Wallich ***Rosaceae*** W: Chyrir / tʃə’rer/ چریر (W: Röhloy/ rrə’lɔi/ غلای) K: Azghan/ азган (K: Eid-muruut/ эид муруут)	160	**Medicine:** Ashes of rose-hips, *röhloy* (W), is mixed with mother's milk and applied to children's ear-ache. **Medicine:** Decoction of rose-hips and dried apricots is drunk for stomach trouble. **Vegetable:** Rose-hips are mixed with barley for nutritional purposes. **Veterinary:** Rose-hips are fed to animals for nutritional purposes. **Fuel:** Fire-wood. **Miscellaneous:** Flowers are used for decoration and infused in warm milk for a pleasant drink.	**Medicine:** Decoction of fruits (rose-hips) is drunk for fever, bloody cough and high blood pressure.	This is one of the few medicinal plants the Kyrgyz admitted to bring from Wakhi territory.
***Rubia chitralensis*** Ehrend. **Rubiaceae** W: Koh-e-rank/ koə’rang/ کوه رنگ	160	**Medicine:** Pieces of root are collected, carried around or kept in households for immediate chewing if inflicted with the potentially deadly bite of the tick *köhna* (W). See text.	Some Kyrgyz know of the Wakhi use, and thank God for not having the tick in the high Pamirs.	Etymology: *koh-e-rank* (W) is a Wakhi adaption of the Persian *rank-i-kui*, meaning 'colour of (the) mountain'.
***Populus pamirica*** Komar **Salicaceae** W1: Reezabark/ ɾeza’baɾk/ ریزه برگ W2: Safeedor/ safe’dɔr/ سفیدار K1: Terik / терик K2: Ak tal/ ак тал	166	**Construction:** General construction, especially the bearing beams in houses.	This tree is well known by the Kyrgyz, however, it does not grow in the high Pamirs, and they do not use it.	Referring to *Populus* with the Dari (Persian) *safeedor* is very common among Wakhi.
***Salix shugnanica*** Görz **Salicaceae** W: Chikor/ tʃkɔr/ چیکار K: Kyrchyn/ кырчын	271	**Construction:** Element in Wakhi layered roof building. **Construction:** Material for baskets. **Fuel:** Firewood.	**Construction:** General construction. **Fuel:** Firewood. **Medicine:** Forty branches blessed by a Mullah may be hung under the yurt-ceiling for curing of various maladies.	The Kyrgyz Kyrchin-valley is named after this shrub.
***Salix spp.*** **Salicaceae** W: Ünük/ wənək/ ونک K: Kyrchin / кырчын K2: Tal / тал	158; 159	**Construction:** General construction and construction of various tools. A number of Wakhi men could easily name ten constructional purposes, e.g. the *anjan* (W), a two-man shovel, brooms, etc.	**Construction:** Indispensable material in the wooden frame-work of the yurt, esp. *wuuk* (K), roof bearing poles, and *keperé* (K), the circular lattice. A type of *Salix* from China is regarded superior.	The Wakhi seem to have a wealth of names for different species, varieties and growth forms for *Salix spp.*
***Scrophularia scoparia*** Pennell **Scrophulariaceae** K1: Kai’ser/ каисар K2: Sary Kai’ser/ сары каисар	201		**Medicine:** Decoction as bath for rashes and swellings, sometimes drunk additionally.	This plant is well known by Kyrgyz, but many people only recognise it by name. It is common in Wakhan, but not in Pamir.
***Hyoscyamus pusillus*** L. **Solanaceae** W: Bang-e-dihwunna/ bangə-dəwɔ’na/ بنگ دیوانه	30	**Medicine:** Smoke of dry seeds is inhaled as a remedy for tooth ache. Small rocks are placed in a dish filled with just so much water that the stones will protrude from the surface. Glowing embers and seeds are placed on the dry rock surface. The smoke emitted is inhaled, under cloth-cover or through a stem or small pipe. Is said to quell the pain. Some informants pointed out this will expell "the worm" from the tooth.		*Bang-e-dihwunna* (W) is a Dari name meaning 'hashish-dizzy' or 'hashish-crazy' which, curiously, in the rest of Badakshan refers to the psychotropic *Datura stramonium* (Solanaceae).
***Myricaria squamosa*** Desv. **Tamaricaceae** W: Tark/ tark/ ترگ K: Bölghön/ бөлхөн	272	**Construction:** Material for screen-door of yurts, often sold to Kyrgyz. **Medicine:** Branches passed quickly through fire, the oil exuded is applied to 'white skin' **Medicine:** The soft, white centre of branches is applied to tooth ache. **Fuel:** Firewood.	**Construction:** Material for screen-door of yurts. **Fuel:** Firewood.	
*Peganum harmala* L. **Zygophyllaceae** W: Schpander/ ’spandər/ سپندر K: Adrashma/ адрашма	1	**Incense:** A soothing incense for sick, upset or frightened people. **Medicine:** Seeds chewed, swallowed with water, for coughing and for 'heart-burn'. **Medicine:** Decoction of seeds drunk for heart trouble. **Medicine:** Seed ground to paste and applied to blister. **Medicine:** Childlessness. Root boiled for approx. 8 hours. The very bitter decoction is drunk on Friday mornings 3 times a month by women having difficulty in conceiving. **Miscellaneous:** Ashes of burned plant mixed and boiled with goat fat to produce soap.	**Incense:** A soothing incense for the sick, upset or frightened people. **Incense:** Incense for headache. **Medicine:** Decoction of seeds for apathy/laziness (depression). **Medicine:** Decoction of seeds drunk when overindulgence in meat and soup has caused discomfort. **Medicine:** Decoction of seeds drunk by pregnant women.	
*Zygophyllum obliquum* Popov. **Zygophyllaceae** W: Yum-wus/ ’jʊm-wʊɛ/ یوم وش K: Burma-qara / булма кара	201	**Vegetable:** Boiled and water discarded, to prevent bitterness, before use in vegetable dishes.	**Medicine:** Remedy for broken bones in human and animal. Dried herb mixed with butter, eaten (or fed) weekly until healed. This herb is known by name and use by many Kyrgyz in Big and Little Pamir, but only recognized on sight by one key informant, who has successfully introduced its use recently.	Etymology: *yum-wus* (W) means 'twin-herb'. *Burma-qara* (K) means 'black drill'.

Vernacular names of the Wakhi (W) and Kyrgyz (K) were confirmed by at least 10 independent sources. The Wakhi names are written in western transcript, Wakhi (modified Persian alphabet) and phonetically. Kyrgyz is written in western transcript and Cyrillic. The column JS# indicates the numbers of the 72 vouchers, in herbaria C (Copenhagen) and KUFS (Kabul), or for non-spermatophyte material; NMM (Museum of Natural Medicine, Copenhagen). The stated uses were confirmed by at least three independent sources

More than half of the plants are represented in two groups or more. The three species that serve the most uses are *Glycyrrhiza uralensis* (fodder, construction material, vegetable, dye, veterinary medicine, fuel), *Juniperus sp.* (medicine, veterinary medicine, construction material, ceremonial use) and *Rosa webbiana* (medicine, veterinary medicine, vegetable, fuel).

Besides the categories in Table 2, some miscellaneous uses should be mentioned. E.g. the Wakhi may mix *Vaccaria grandiflora* into grain before milling to produce whiter flour. Wakhi women considered a few plants good fermentation-starters in yogurt-production: *Cicer microphyllum* (preferred), *Ephedra intermedia* and *Oxyria digyna.* Oil from the seeds of *Brassica napus* and juice pressed from *Artemisia persica* is applied to face and hands specifically to shun the coldness of the wind. *Juniperus sp.* is used in the ritual of carrying branches into and around houses of neighbours and relatives during the social visits associated with Nowruz (New Year), on account of it being an ever-green. Oil pressed of seeds of *Brassica napus* was formerly used in lamps as the sole light-source in Wakhi homes. With the introduction of solar panels and car batteries this practice has gone out of use, but pressed oil from *B. napus* is still used for medicinal purposes.

#### Crops and commercial plants

The Kyrgyz, being nomadic, have no crops. They barter or buy some crop plant products namely wheat (*‘buddhai*’) in the form of flour (‘*un*’), barley (‘*arpa*’), rice (‘*gürüch*’), vegetable oil (‘*mai*’), tobacco (*‘tumakuh*’) in the form of cigarettes (‘*paprus*’) and snuff (‘*naswe*’, made from *Nicotiana rustica* grown in Wakhan) and opium (‘*tarii*’*ak*’).

In Wakhi fields and gardens within the study area is grown wheat *Triticum aestetivum* (‘*gedim*’), barley *Hordeum vulgare* (‘*yirk*’), millet *Panicum miliaceum* (‘*yirsen*’), grass pea *Lathyrus sativus* (‘*kross*’), pea *Vicia faba* (‘*sach*’), bean *Pisum sativum* (*‘baqla*’), hemp *Cannabis sativa* (‘*chars*’), tobacco *Nicotinia rustica* (‘*tumakih*’), potatoes *Solanum tuberosum* (‘*kachalu*’) and caper *Capparis spinosa* (‘*kaper*’). A few apricot trees (*Prunus armeniaca*) and apple trees (*Malus domestica*) are found in villages. Plots of poplar (*Populus sp.*) and willow (*Salix sp.*) are planted for fuel and construction material. Rapeseed, *Brassica napus,* abounds in fields, which in the spring turn completely yellow on their account. Other authors count it as a crop [6], but Wakhi respondents claimed it is no longer sown, and simply occurs spontaneously. The leaves of *B. napus* are used extensively as a vegetable in the spring. Caper supposedly is a rather recent introduction as crop, but grows wild or naturalised in Wakhan. Potato was stated by a number of respondents to have been introduced to Wakhan in the 1980’s by Russians during the Soviet occupation of Afghanistan.

#### Fuel

Fuel for warmth, cooking, etc., is a scarce resource in the Wakhan and Pamir and it is a considerable task to acquire enough to meet household needs. For the Kyrgyz in the Pamirs, the only wooden fuel sources are *Artemisia*-shrubs, *Krascheninnikovia ceratoides* and *Acantholimon spp.* The Wakhan valley is comparably gifted with a number of firewoods (e.g. *Salix spp., Hippophaë rhamnoides, Rosa sp.* and even *Ribes sp.*), but not in abundance. The fragrant *Juniperus-*wood has become so rare it is only used as firewood for the most special of occasions. In Kyrgyz households, and in Wakhi Pamir pastures, wooden shrubs serve mainly as a kindle for yak-dung, compressed sheep dung and the occasional cut peat.

#### Fodder

To feed animals throughout the winter it is necessary for both Wakhi and Kyrgyz to collect fodder. This mainly consists of cutting and drying grass and sedges. Other plants collected include *Artemisia dracunculus, Chenopodium spp*. and *Glycyrrhiza uralensis,* among others. The legume *Cicer microphyllum* is a wild fodder plant much valued by both Wakhi and Kyrgyz. Plants sought out by herders for grazing were not focused upon in this study.

#### Construction

Wood is the principle material for construction, and a scarce resource. The Kyrgyz parts of Pamir are well above the tree-line, so all construction materials (if not animal bone, tooth, skin, wool or hair), must be transported up from lower altitudes, except from the gnarly *Salix*-shrub and the occasional piece of metal or larch-wood left behind by the Soviet military outposts. Most of the Kyrgyz wood-materials are *Salix* and *Betula,* which are mainly taken from the small riverine forests in lower Pamir. Kyrgyz tools, spindles, whips and nose-woods for animals are often *Lonicera spp.* collected in lower Pamir or Wakhan, which is true also for long, strong grasses (esp. *Calamagrostis pseudophragmites*), used for room-dividing and insulating screens in the yurt, as well as for platforms to dry various dairy products. Grass-screens and screen-doors (from *Myricaria sp.*) made by Wakhi are bartered. The scarcity of construction material is felt especially in high Pamir where any random piece of wood is picked up for use, especially if big enough for mooring of horses, currcomb, fire-poker and other bric-a-brac.

Wakhi have considerably more constructional resources than the Kyrgyz: *Populus, Salix, Betula* and *Hippophaë rhamnoides* are the principle ones. Wakhi men especially can name a number of varieties of both *Salix* and *Populus* and their uses according to their properties. A notable example is for the most flexible of *Salix* to be used in the ‘*an-jan*’, the two-man-shovel for digging irrigation canals. *Betula*-wood is indispensable in the construction of the plough and oxen neck-piece (*‘svör* and *‘spunder*’). Baskets and other household objects are often made from slender *Salix*-branches and long, strong grasses such as *C. pseudophragmites.* For finer woodwork and for dowels (wooden nails*) Lonicera sp*. is used. *Lonicera* is also the preferred wood for the ubiquitous wool-spindles.

The Wakhi house’s three most prominent wooden features are the 5 carved pillars and the bearing beams on top of them, and the uniform quadrangular pattern of heavy beams framing the smoke-hole. The pillars and beams are cut into shape from trunks of *Populus,* although *Juniperus* was traditionally preferred. The more or less flat roof consists of layers of materials laid perpendicular for each layer, starting with *Salix* poles on top of the *Populus*-beams, and followed by layers of medium sized branches of e.g. *H. rhamnoides* and *Salix*, an insulating layer of e.g. *Glycyrrhiza uralensis* or *Artemisia*-shrubs, and a layer of grasses such as *Phragmites australis* or *C. pseudophragmites* before being covered by a layer of ‘mud’ (*löb*). Few or none metal objects are used in Wakhi houses, which may reach considerable age. Other construction such as pasture huts, herders’ shelters, stables and outhouses are less elaborate and often constructed with *Betula, Salix* or *H. rhamnoides,* mud and stone*.*

The Kyrgyz yurt consists of a wooden frame-work, yak hair robes, straps and felt covers. The crown of the Kyrgyz yurt, called *tündök*, is made from *Betula*. The circular lattice work (*kepere*) and the roof poles (*wuuk*) are made from *Salix*, as informed by the Kyrgyz yurt-craftsmen using the Dari words *har-beet* and *sar-beet*: ‘black’ and ‘yellow’ willow. The identity of the wooden elements was confirmed by anatomical studies of wood samples taken from yurts in Big and Little Pamir [14]. Elder Kyrgyz mentioned that a superior, stronger wood was formerly imported from Kashgar in China before the closing of the border in 1949. This wood is called *kara-sögod* and *sara-sögöd*. Wood samples taken from a very old yurt in Little Pamir indicated that this wood indeed may be stronger on account of the conspicuous heart-wood, which no trees in Wakhan appear to have at the appropriate age for gathering. Anatomy showed that this wood was also a species of *Salix* [14]*.*

Recently the Wakhi have started using yurts on their pastures. These are usually bartered from the Kyrgyz, but a few Wakhi have begun making yurts themselves and experiment with new materials.

#### Cosmetics

Cosmetics in Wakhan and Pamir are almost entirely focused on skin moisturizing, with the exception of black eye-liner and rouge made by Kyrgyz women from the red dye of the root of *Arnebia guttata*. Wakhi men and women use a species of *Delphinium,* which is heat-extracted into oil (fried) for dry hair and dandruff. Skin moisturizing, especially in Wakhan, is almost in the realm of medicine, as many people are suffering from extremely dry and cracked skin, especially their hands and faces. This is caused by the dryness of the air, near constant wind and outdoors manual labour under high UV-radiation. For the most severe cases of skin cracks a paste is made from the flowers of *sart-sprech* (meaning ‘yellow-flower’), a plant name that includes many species of both *Ranunculus* and *Potentilla*. One key respondent said that the correct flowers to use are the soft and delicate *sart-sprech* near streams, indicating herbaceous *Ranunculus* instead of the sturdier *Potentilla*-species. *Sart-sprech* is the only skin moisturizer men would admit to use. Wakhi women make moisturizer from berries of *Lonicera pamirica*, *L. asperilifolia* and *Hippophaë rhamnoides*, the latter of which is also said to have sun-protecting qualities. Moisturizer and facial crèmes are furthermore made from flowers of *Primula pamirica* and *Myosotis asiatica*, and the sap pressed from sun-heated *Elsholtzia densa* and the oil pressed from seeds of *Brassica napus*.

#### Dyes

Natural dyes for wool and clothing among Kyrgyz only include *Arnebia guttata* root, which produces a strong red colour. *A. guttata* is also used by Wakhi, as well as *Glycyrrhiza uralensis* and *Artemisia persica,* both producing yellow colour. Both Kyrgyz and Wakhi women wear colourful clothes, but they are mainly made from pre-dyed fabrics. Colourful wool-crafts in Wakhan, especially socks and gloves, are now made with chemical dyes. Some natural dyes made from local minerals can be seen in both houses and yurts, but this practice appears to be at the verge of dying out.

#### Vegetables

The Kyrgyz use wild onions *Allium spp.* as a cooked vegetable often with meat. This apparently constitutes the only prepared vegetable in their diet. All other vegetables are consumed fresh and raw. These includes roots (*Polygonum viviparum*), leaf stalks (*Rheum spiciforme*), leaves (*Oxyria digyna*), berries (*Ribes villosum, Ephedra regeliana*), flowers and seeds (*Cicer microphyllum*). A common delicacy among Kyrgyz is to serve the seeds of *P. viviparum* with thick cream.

The Wakhi have a strong tradition for collecting wild vegetables, both for immediate use and for drying and keeping. They consume the same fresh roots, berries, leaf stalks, leaves, flowers and seeds as the Kyrgyz with the addition of fruits of *Rosa webbiana*, the seeds of *Carum carvi* and *Silene conoidea*, and the rather rare, occasional chewing of *Glycyrrhiza uralensis* root.

The main use of vegetables however is for a dish called *rasch. Rasch* is a mixture of one or more green vegetables, fried in oil, and often with salt, milk and flour added. Some herbs are first boiled, and the water discarded, due to bitterness. Other herbs, especially the unidentified *lach,* may be boiled before curled into balls and dried for later use. *Rasch* is an element of every hot meal throughout the spring and early summer. The plant-names presented in Table 4 were cumulated from the first two group interviews, and probably not exhaustive; Wakhi *rasch* plants could easily serve as a study by itself. Compared to most men, women are considerably more knowledgeable on plants useful as *rasch*-vegetables.

**Table 4:** Plants used for *rasch* by the Wakhi

Plants used for ‘*rasch*’	
Shög-shög, Katch,	*Allium spp.*
Lönntörk, Tuk-lönntörk	*Allium spp.*
Cherok	*Brassica napus*
Schlitt	*Chenopodium spp.*
Gudunch wus	*Elsholtzia densa*
Ziuck	*Chenopodium botrys*
Yum wus	*Zygophyllum obliquum*
Schellkhar	*Rumex sp.*
Nurtök	*Carum carvi*
Paps	*Taraxacum sp.*
Lach	-
Yürk wus	-
Kebit wus	-
Khöröv	-
Svartjal	-
Sjars wus	-
[…]	

#### Tea

Black and green tea is, as in most of Asia, a prominent feature of everyday life. Although green tea is enjoyed in Wakhan and Pamir, the numerous cups drunk daily by men, women and children are made from black tea, made by decoction (a few minutes of boiling), and copious amounts of milk and/or cream. Preferred milk is from goat and sheep, followed by yak and cow, but in both Kyrgyz and Wakhi custom these are usually mixed. Salt is added, either in the form of commercial salt, or local salt rocks crushed and dissolved in water.

Tea-substitutes are numerous but, unless in a medicinal context, rarely used as long as real tea is available. They are prepared in the same way as the milk-tea and include in Wakhan *Artemisia persica*, *Chenopodium botrys*, *Elsholtzia densa*, *Ziziphora clinopoides*. In Pamir, the tea-substitutes are *Dracocephalum paulsenii*, *Nepeta pamiriensis* and *Z. clinopoides.* The perhaps best-known tea-substitute among both Wakhi and Kyrgyz is *Potentilla bifurca.* It is collected in the fall, after flowering, when the leaves are curled up and the entire herb has a characteristic red colour.

#### Incense

Is mainly used in a medicinal context, as a soothing or calming agent. Incense is made by burning fresh or dried *Juniperus sp, Peganum harmala* and, less commonly, *Anaphalis virgata.*

#### Veterinary medicine

More than 20 % of the use-plants have a veterinary use, testifying to the Wakhi and Kyrgyz dependence on their animals’ well-being, workability and survival. The most common veterinary use is plants for wounds, particularly maggot-infested wounds. It is common for pack animals to get chafes; wounds of wear. Especially horses often have chafing scars on their upper back. Wakhi will apply dry *Rumex*-root, ground *Delphinium* sp. or *Artemisia persica* on the wound. The Kyrgyz may use the spores of mushrooms, especially that of puff-ball, *Calvatia* sp., to “dry” the wound, and the Kyrgyz of Little Pamir state that *Pulsatilla campanella* is an effective antiseptic and/or wound healer. For broken bones in sheep and goats, the Kyrgyz, mainly in Big Pamir, feed them dry *Zygophyllum obliquum* mixed with butter. Both Wakhi and Kyrgyz use lichens as veterinary medicine in various ways. In springtime, when many animals suffer from diarrhoea, which can be life-threatening especially for yak-calves, they are fed lichens, grounded and mixed in butter. In Wakhan, the large herb *Tetranum olgae* causes blindness to animals that eat it, which is treated by rubbing the eyes with coal of *Juniperus* sp. Calves suffering from ‘sunburn’ with blisters on their back are in Wakhan fed *Mentha longifolia*. Calves with constipation are fed the oil of *Brassica napus* seeds. *Glychyrrhiza uraliensis* is fed to nursing cows to increase their milk quality. Rosehips are fed to animals for nutritional purposes. Oxen and horses are fed a decoction the root of *Berberis heterobotrys* as an anthelmintic and as a strengthening remedy when they are needed for intense labour in the spring, but are weak after a long winter.

#### Traditional medicine in Wakhan and Pamir

Duncan & Duncan [8] found that 70 % of Wakhi use ‘local remedies’ for treating illnesses. In the present study was recorded 32 plants used medicinally by the Wakhi. The Kyrgyz use 15 plants medicinally, which constitutes 40 % of their total number of use-plants. The most common disorders for which medicinal plants are used are summed up in Table 5. Details on indications, preparation and administration are in Table 3. Both among Wakhi and Kyrgyz, medicinal plants are collected and used when needed, as well as collected and dried for either later or regular use. In most cases, preparations of remedies are made with just one plant, mixed remedies being less common. The primary form of preparation is by decoction, as with traditional tea, by boiling the plant material for several minutes. Infusion is less common. The preference to decoction over infusion can possibly be explained by the lower boiling point of water in the high altitudes. External application of either dry herbs or paste/poultices is also common. On top of this, especially among the Wakhi, there is a number of distinct methods of preparation and administration. Neither among Wakhi or Kyrgyz there appears to be any ‘protectionism’ in regard to traditional medicinal knowledge, and knowledge is freely shared and discussed at ease. However, certain Wakhi families are locally renowned for being especially knowledgeable. Except for opium and a remedy called ‘sechestaband’ (used for broken bones in man and animal) all traditional medicines stems from local plants.

**Table 5:** Most common uses of medicinal plants

	Wakhi – no. of uses	Kyrgyz – no. of uses
Wounds, burns	5	
Dermatology	3	3
Skin moisteners	7	
Eyes – throat – ears - teeth	10	
Respiratory problems	4	1
Fever	7	5
Cold	2	
Headache	3	3
Joint pains, back pain	4	1
Broken bones	2	1
Stomach – gastro-intestinal	10	2
High blood pressure, heart problems	6	5
Swelling	6	
Incense - mental	3	3
Jaundice	2	
Altitude sickness	1	2

#### Kyrgyz uses of medicinal plants

The most common disorders treated with plants among the Kyrgyz are fever (5 plants), headache (3 plants), high blood pressure and heart-trouble (5 plants). Rashes and swelling (3 plants) are often treated with baths. Altitude sickness (2 plants) are also among the disorders treated traditionally, as are psychological inflictions such as ‘angst/anxiety’ and depression (3 plants). The Kyrgyz often complain of “pain”, for which smoking of opium is readily used. Many Kyrgyz stated that drinking milk is a principle factor for maintaining good health. The by far most common way of preparation is by decoction. Infusion is apparently never used among the Kyrgyz. As with tea, milk is often added before drinking the plant decoction. Use of the aromatic *Anaphalis virgata* for high blood-pressure is highly valued by many Kyrgyz men, who stressed that it should be prepared by ‘double-decoction’. Decoctions used for bathing or washing may be simultaneously drunk, and include those of *Allium sp.* and *Artemisia persica* for itching rashes and swellings. *Peganum harmala* is the plant with most medicinal uses among Kyrgyz. It is especially valued as soothing incense for the sick or frightened. Ointments or pastes are not widely used among Kyrgyz, although a few mentioned an ointment made from *Delphinium sp*. applied to cuts and wound, and that a paste made from the green parts of *Ephedra intermedia* rubbed on the stomach, chest and shoulders is used against ‘fear’.

#### Wakhi uses of medicinal plants

The primary disorders for which the Wakhi use medicinal plants are fever (7 plants), heart trouble and blood pressure (6 plants), gastro-intestinal problems (10 plants), jaundice (2 plants), headache (3 plants), joint pain and back pain (4 plants), swelling (6 plants), respiratory problems (4 plants) and children’s ear-ache, burns, blisters, abscesses and wounds (10 plants). The most versatile medicinal plants in Wakhan are in general also the most valued, based on statements from interviews. *Peganum harmala*, *Rumex spp*., *Ephedra intermedia*, *Plantago sp*., *Elsholtzia densa*, *Nepeta pamiriensis* and *Ziziphora clinopoides* all have several medicinal uses and are among the most valued medicinal plants. *Berberis heterobotrys*, used for high blood pressure and heart-trouble, is another valued remedy and fruits are often picked by Wakhi for relatives who do not have easy access to the plant.

The most common form of administration is by drinking decoctions of herbs, leaves, seeds or root, often with milk added. Some notable remedies made by decoction are those of aromatic plants like *Anaphalis virgata, Artemisia persica, Elsholtzia densa, Mentha longifolia, Ziziphora clinopoides* and seeds of *P. harmala*. Many of the essential oils in these plants have been identified from material collected in Wakhan and Pamir [15]. A liquid made from maceration and straining of fruits of *Berberis heterobotrys* is bottled and preserved with sugar for regular (daily) use, as is a liquid prepared from fruits of *Hippophaë rhamnoides* drunk for joint pains (gout). Ointments and pastes are common especially for leg pain, swellings, blisters and wounds (e.g. *A. persica, Epilobium latifolium* and *Plantago* sp.). A bath for swellings and rashes is made from *Allium sp.* and *Ephedra intermedia*. A “decoction-bath”, especially known among men, is that of *E. intermedia* into which a broken or dislocated limb is submerged while being relocated. Simply eating medicinal plants is also fairly common, e.g. *P. harmala* seeds for a variety of ailments. Incenses from especially *Delphinium sp*., *P. harmala* and *Juniperus* sp. are considered soothing for the sick. For dental hygiene, the use of the cleaned root of *Astragalus sp.* as a toothbrush is wide-spread in Wakhan. A mouthwash is made by decocting *E. intermedia*. Though opium smoking has been largely abandoned in Wakhan in recent times, it is still considered a powerful medicine, especially in pain-relief. However, opium is by some regarded an omnipotent medicine, and there are examples of opium being used in small, even symbolic, amounts, in combination with other medicines. Other medicinal plant prepations include inhaling the smoke of *Hyoscyamus pusillus* seeds, blowing grounded seeds of *Descurainia sophia* into a sore throat, burning of dry scrapings of *Rumex sp*. on skin (as in East Asian moxibustion), rubbing the white ‘dust’ of *Primula macrophylla* into the eyes, wrapping fever patients in wet leaves of *Rumex sp.*, and the chewing of the root of *Rubia sp*. as an antidote to the life-threating bite of a tick (see below).

Medicinal plants are often prepared as a dish known as *rasch,* which is mostly collected and prepared by women. It is made by chopping fresh or dried green plant material, frying it in butter and oil and usually milk, cream, flour and salt, and served warm. It is a significant element in Wakhi traditional medicine, and it is notable that, especially women, will mention *rasch* as the first thing when asked about medicinal plants. Furthermore, Wakhi women are hesitant to distinguish between food and medicine when it comes to *rasch,* and it is considered essentially to good health. However, “medicinal *rasch*” may be prepared for specific ailments. The season for ‘vegetable *rasch*’ is spring and early summer where intake of vitamins after a long winter is doubtlessly important, and in these seasons it accompanies every hot meal. ‘Medicinal-rasch’ may be prepared with just one medicinal herb, or by adding it to the mix of plants normally used for the ‘vegetable-*rasch*’. A list of some of the plants used in *rasch* is provided in Table 3. *Rumex sp.* leaves, *Carum carvi* herb and *Allium sp*. and *Mentha longifolia* are used in *rasch* against fever. The *Allium sp,* known as *lönntörk* is used in *rasch* against diarrhoea. *Plantago sp.* leaves are used against constipation. A less common use is *Rhodiola heterodonta* leaves against ‘accumulation of blood’.

A tick known as *könah*, identified as *Ornithodoros lahorensis*, is considered potentially lethal by the Wakhi. Respondents would tell it lives in old houses and that people from time to time are bitten in their sleep. Symptoms include rashes on the chest and other parts of body and swelling of the tongue, which is said often to lead to asphyxiation and death. These symptoms were confirmed by one respondent who survived an incident. Although the swelling of the tongue restricts speech, the condition is so well known that family members will react promptly: The tick is caught, thrown on the fire place embers for the victim to inhale the smoke. Next, the victim should vigorously chew the root of the plant known as kui-rank, or rank-e-kui, in Wakhi and Dari respectively. The plant was identified as *Rubia sp.* (*R. chitralensis* and/or *R. tibetica*). Finger-sized pieces of root are found in many house-holds, and when asked, some Wakhi would produce a piece from a pocket, tied to a waist-coat or sown into a coat. At least some respondent mentioned that if opium is available, the victim should hold a small piece of opium under the tongue while chewing the root. Finally, the victim should be immobilized and continuously over-poured with cold water. The symptoms are coherent with the condition of acute anaphylactic shock. An antihistamine assay on guinea-pig ileum using material collected in Wakhan supports this use of *kui-rank* [16]*.*

An in-vitro study of the antibacterial and Cox-1 inhibitory effect of plants used to treat inflammatory ailments, pain, fever and infections, supported some of the local uses of *A. persica, Dragocephalum paulsenii, E. intermedia, H. pusillus, Lagochilus cabulicus, Nepeta parmiriensis, P. harmala, Rumex patientia subsp. pamiricus,* and especially that of *Arnebia guttata*, which is used for children’s ear-ache, usually caused by bacterial infections and inflammation [17].

#### Origins of plant resources

The Afghan Kyrgyz nomads collect a range of wild plants both from areas near and around their seasonal camps in the high Pamir, as well as from areas of considerable distance in lower Pamir and Wakhan. In fact, the Kyrgyz find some 15 % of their use-plants in Wakhan, which for all Kyrgyz is at least one, but more likely several days’ journey. When the Kyrgyz gather useful plants in Wakhan they only take truly wild plants and are not familiar with any of the useful anthropogenic plants in and around Wakhi villages and fields. Only one use-plant appears to be an indirect result of their presence in the Pamirs; a conspicuous *Chenopodium sp.* which is apparently only found in the disturbed soil around Kyrgyz winter camps. It is burned and used as potash in soap production. Plant exchange with the outside world consists mostly of agricultural products (mainly flour and rice), which the Kyrgyz either barter with livestock or receive as food aid. The Kyrgyz also barter considerable amounts of opium for local consumption.

The Wakhi identified their main areas of plant resources to be 1) the environment of their irrigated fields and villages, 2) the unirrigated surroundings of their villages (2800–3300 m.a.s.l.) and 3) on route to or in their pastures in the Wakhan Hindukush, Big or Little Pamir. Though the primary function of the pasture is as an indispensable grazing-ground, the Wakhi also perceive it as a resource of useful plants especially for wild vegetables and medicinal plants. The environment of the irrigated village and surroundings supply the Wakhi with 22 % of their useful plants, almost all of which cannot be found outside the fields and village grounds. When botanist Ove Paulsen visited Wakhi villages in 1899 he noted ‘*Weeds abound everywhere in the cultivated land. The fields present a sorry spectacle indeed* […]’and listed 27 species of so-called ‘weeds’ [11]. Still today, the Wakhi do not practice weeding of their fields, but this study showed that they collect and use at least nine of Paulsen’s ‘weeds’.

The Wakhi are self-sustainable in most basic staples except rice. A few plant products are either bought or bartered in the Iskashim bazaar or from itinerant traders. These are mainly spices such as pepper, cardamom and turmeric, and a medicinal remedy called *sechestaband*. Although there is a demand in Badakshan for the roots of *Glycyrrhiza uralensis* [18], which is very abundant in Wakhan, and for the asphalt-like substance called *mumion* or *salajeet,* which allegedly is found in certain caves in Wakhan and Pamir, neither of these are brought to market.

#### Plant knowledge among Wakhi and Kyrgyz subcultural groups

Two distinct Wakhi pastoral traditions exist: there are those who take their animals to the Big Pamir in the summer, and those who use pastures in the relative vicinity of their village in the Wakhan Hindukush. When only considering plants growing in or near Wakhi villages (on the scale 1–3; more or less common within half a days travel from habitation) the average recognition rate of these use-plants were for both Wakhi groups around 80 %. However, when considering all the “Wakhi use-plants”, the Hindukush-pastoralists recognised 70 % and Big Pamir-pastoralist averaged 79 %, the difference being mainly made up by high-altitude plants. This could be explained by the Big Pamir-pastoralists moving over greater distances and to higher altitudes and thus having more exposure to high-altitude plants.

The Kyrgyz constitute two well-defined groups; those who live in Big Pamir and those who live in Little Pamir. Both had an average recognition rate of the “Kyrgyz use-plants” of 45 %. Some minor differences between the two subcultural groups were observed. *Pulsatilla campanella,* which is used as a veterinary medicine in Little Pamir is completely unknown in Big Pamir. And, the other way around, one of the most used medicinal herbs in Big Pamir, *Ziziphora clinopoides,* is hardly known in small Pamir, despite comparable availability.

#### Plant use and knowledge compared by gender

Individual interviews with Wakhi men and women revealed no significant difference in the total number of plant names and uses known to either gender. However, the specific plants and their uses varied to some extent between the genders. There was a tendency for Wakhi men to be more knowledgeable of the grazing and fodder plants than women, and for women to know more domestic uses of some plants than men. Very few Wakhi women have travelled in Kyrgyz parts of the high Pamir, and they are rarely responsible for herding animals at high altitudes. This was reflected in women’s lesser ability to recognise high-altitude plants.

Kyrgyz women are often not allowed to speak or be alone in the presence of strangers. Thus individual interviews with Kyrgyz women were rarely permitted. Furthermore, members of both genders stated that men would be considerably more knowledgeable on plants, and interviews with women therefore would be pointless. This was however contradicted in an interview with a 70-year old widow, who proved able to apply names and uses on more plants than any Kyrgyz male. The total five interviews with Kyrgyz women did not provide basis for conclusions on gender-specific plant knowledge.

#### Inter- and intracultural exchange of plants and plant knowledge

Exchange of mainly medicinal plants and plant knowledge was observed to be common between various Wakhi subcultural groups. Many Wakhi will ask relatives for whom a certain plant is more available to collect it for them. This is especially true in the case of medicinal and veterinary *Berberis heterobotrys*, both the root and the fruit, and with the fruits of *Capparis spinosa.* Often, when a certain plant is not available around a Wakhi village, the inhabitants will still know the nearest place to acquire it. For example, in mid-upper Wakhan everyone on the southern bank of the Wakhan River knows *Mentha longifolia* only grows on the northern bank (half a day’s journey away).

Intra-cultural exchange appeared limited among the Kyrgyz geographical sub-groups. For example, almost no one in Little Pamir knew of any medicinal use of *Ziziphora clinopoides,* which in Big Pamir is used for high blood pressure and other circulatory troubles. Apart from some collection and exchange of *Zygophyllum obliquum* among Kyrgyz in Big Pamir, most Kyrgyz do not seem to buy, barter or exchange any wild medicinal plants.

Exchange of plant products between the Wakhi and Kyrgyz occurs to a smaller extent than what could be expected in such a confined environment as Wakhan and Pamir, and is limited to a few agricultural products. Marriage between Wakhi and Kyrgyz is unheard of, and this greatly limits integration of knowledge between them. Their respective plant vocabularies did not suggest exchange of knowledge. In no case did Wakhi and Kyrgyz use the same name for a plant, although they might be knowledgeable of the others’ names, or both know the Dari name. Nonetheless, the Kyrgyz and Wakhi have many common plant uses.

Thirty-three species of plants have at least one similar use among both Wakhi and Kyrgyz. Most of these plants are essential to survival in environments of Wakhan and Pamir and would be identified for use by any group with or without knowledge exchange. These include all the fuels (*Artemisia spp.*, *Krascheninnikovia ceratioides, Acantholimon spp., Salix spp.*) and the construction materials (*Salix spp., Betula sp., Myricaria squamosa*, *Calamagrostis pseudophragmites, Lonicera spp*.), as well as the grazing plants and fodder plants (mainly species of Poaceae, Cyperaceae and Fabaceae). However, there are some candidates for intercultural knowledge exchange, such as the similar uses of wild vegetables such as *Allium sp*., *Ribes*, *Rumex spiciforme*, *Oxyria digyna, Cicer microphyllum* and *Polygonum viviparum*, and the common medicinal uses of *Juniperus* and *Peganum harmala.* The two latter are however well-known across Central Asia. The best candidates for intercultural knowledge exchange are the Kyrgyz use-plants only found in Wakhan, and the Wakhi use-plants only found in the high Pamir. For example, the medicinal uses of rose-hips and the fruits of *Berberis heterobotrys*, which are well-known to most Wakhi, are also known and used by at least some Kyrgyz, although these plants do not grow in the high Pamir. The other way around, the medicinal uses of the aromatic plants *Anaphalis virgata* and *Dracocephalum paulsenii* are well-known to many Kyrgyz, but only to those Wakhi who have pastures in the Pamirs near the Kyrgyz. Both the Kyrgyz and Wakhi expressed much interest in each other’s plant uses. When the expedition returned to Wakhan from Pamir, the news of Kyrgyz medicinal and veterinary use of *Zygophyllum obliquum* spread quickly among Wakhi, who until then only knew the plant as a vegetable.

#### Comparison of Wakhi and Kyrgyz ethnobotany

The Wakhi have a use-plant inventory of 62 wild plants or plant groups, while the Kyrgyz have an inventory of 37 wild plants or plant groups. Due to the relatively lower altitude, the Wakhi have more plants growing in the vicinity of their settlements, and their irrigated fields and villages furthermore allows the existence of many species which are otherwise not found in Wakhan. Twenty-two percent of the Wakhi use-plants are anthropogenic and an indirect result of their land-use. However, even when the plants that occur due to agricultural irrigation are excluded, the lower altitude (2700–3100 m.a.s.l compared to the Kyrgyz’s +4000 m.a.s.l.) facilitates a considerably greater biodiversity available to the Wakhi.

A comparison of the distribution of plants in the 10 use-groups (Table 2) shows that the Wakhi use roughly twice as many plants within each group. Exceptions from this pattern are fodder plants in which the Wakhi and Kyrgyz were both noted for 6, and cosmetics in which the Wakhi were noted for 10, the Kyrgyz only for one plant. Most of the Wakhi cosmetics were skin moisturizers, which the Kyrgyz apparently do not use, despite the even harsher climate in the high Pamirs. The largest use-group, within both cultures, are the medicinal plants. The Wakhi use 32 plants medicinally, whereas the Kyrgyz only 15. Furthermore, the Wakhi have a much wider range of preparation and administration, where the Kyrgyz mainly utilize decoction and external administration (ointments etc.). The most common maladies that the remedies address are comparable, mainly fever, pain, heart-trouble, blood pressure, blisters and wounds (Table 4) but on top of this the Wakhi also address more specific maladies such as jaundice, children’s ear-aches and respiratory problems. The only area in which the Kyrgyz exceeds the Wakhi (4 plants to 3) are in that of psychological disorders (‘fear’, unrest, anxiety and depression).

The use-plants which are obtained furthest away are among the Wakhi *Salix shugnanica* used for weaving baskets, *Zygophyllum obliquum* used as a vegetable, and *Primula macrophyllum,* which is valued eye medicine. Among the Kyrgyz, the most distant use-plants include the medicine and veterinarian *Z. obliquum,* the medicinal *Anaphalis virgata* and *Peganum harmala,* and especially the indispensable constructions materials of *Calamagrostis pseudophragmites,* S*alix sp*. and *Betula sp*. Both the Wakhi and the Kyrgyz use *Juniperus sp.,* for a variety of purposes, but according to respondents, less so in recent years due to depletion. Likewise, good *Betula sp.* is becoming rare in the Wakhan Corridor and its depletion could be a serious threat to Kyrgyz traditional yurt-craftsmanship.

**Fig. 3:**
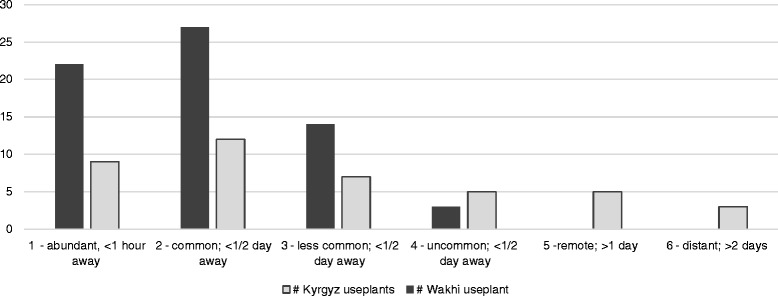
Availability of use-plants among Wakhi and Kyrgyz

Figure 3 shows the number of respective Wakhi and Kyrgyz use-plants within each availability level. For both Wakhi and Kyrgyz there is an obvious tendency to find a decreasing number of use-plants further away from their respective habitations. However, also apparent from Fig. 3 is the fact that the Kyrgyz find a greater proportion of their total number of useful plants considerably further away from their habitation than the Wakhi from theirs.

It is tempting to interpret the difference in the proportion of use-plants in relation to the distance from the two peoples’ respective habitations as an inherited function of their two distinct livelihoods. That is, ‘the nomads’ are more mobile and cover greater geographical areas than ‘the sedentary farmers’, and thus, despite the fact that they live in a more plant-deprived environment, their nomadic livelihood exposes them to useful, although distant plants. It is however rather more likely that these patterns reflect necessary adaptions to an extreme environment, where procuring the necessary materials for survival (fuel, construction material, vegetables, medicine, etc.) comes at a considerable cost of labour, time and transportation. Life in the comparatively plant-deprived environment of the high Pamirs, more than 4000 m.a.s.l. has necessitated the Afghan Kyrgyz nomads to develop knowledge and uses of plants, which are considerably remote to them, when compared to their closest neighbours, the Wakhi, who enjoy a relative richness of biodiversity, down at 2700–3300 m.a.s.l. The difference in efforts that these two peoples endure to obtain life-sustaining plant resources appears to be accentuated by environmental conditions at the cusp of what is humanly possible.

## Conclusions

The present study for the first time documented a wealth of endemic, indigenous plant knowledge; from unique plant name etymology to highly specialized medicinal plant uses, in the geographically extreme and isolated Wakhan and Pamir of Afghanistan. Although the agropastoralist Wakhi and the semi-nomadic Kyrgyz are close neighbours, in a confined area, there are striking differences in their adaptations to their environment, as reflected in their plant use and knowledge. Overall, the Wakhi have a greater biodiversity available to them, and utilize more plants than the Kyrgyz. Wakhi traditional medicine also proved to be considerably more complex, with a greater number of remedies for a greater array of maladies, and a greater range of methods of preparation and administration. Although Wakhi land-use contributes to the greater biodiversity available to them, it appears to be the difference of the environments, especially the 1000 altitudinal meters that separate the Wakhi and Kyrgyz settlements, that has necessitated the Kyrgyz to develop knowledge and uses of plant resources which are comparatively remote to them, in order to survive in the high Pamir. Despite the striking differences between the two neighbours, there is for the visiting researcher one trait the people of Wakhan and Pamir have in common; an exceptional forthcomingness and willingness to share both their scarce resources and their knowledge of plants; undoubtedly another admirable adaptation to an extreme environment.
